# Free Flap Transfer to Preserve Main Arterial Flow in Early Reconstruction of Open Fracture in the Lower Extremity

**DOI:** 10.1155/2015/213892

**Published:** 2015-03-12

**Authors:** Mitsuru Nemoto, Shinsuke Ishikawa, Natsuko Kounoike, Takayuki Sugimoto, Akira Takeda

**Affiliations:** Department of Plastic and Reconstructive Surgery, Kitasato University Hospital, 1-15-1 Kitasato, Minami-ku, Sagamihara, Kanagawa 252-0374, Japan

## Abstract

The selection of recipient vessels is crucial when reconstructing traumatized lower extremities using a free flap. When the dorsalis pedis artery and/or posterior tibial artery cannot be palpated, we utilize computed tomography angiography to verify the site of vascular injury prior to performing free flap transfer. For vascular anastomosis, we fundamentally perform end-to-side anastomosis or flow-through anastomosis to preserve the main arterial flow. In addition, in open fracture of the lower extremity, we utilize the anterolateral thigh flap for moderate soft tissue defects and the latissimus dorsi musculocutaneous flap for extensive soft tissue defects. The free flaps used in these two techniques are long and include a large-caliber pedicle, and reconstruction can be performed with either the anterior or posterior tibial artery. The preparation of recipient vessels is easier during the acute phase early after injury, when there is no influence of scarring. A free flap allows flow-through anastomosis and is thus optimal for open fracture of the lower extremity that requires simultaneous reconstruction of main vessel injury and soft tissue defect from the middle to distal thirds of the lower extremity.

## 1. Introduction

Reconstruction of a traumatized lower extremity using a free flap carries a greater risk of developing complications compared to reconstruction of other sites [1–4]. Many investigators have reported the importance of selecting an appropriate recipient vessel when reconstructing the lower extremity by free flap transfer [5–7]. To enhance the success rate of free flap transfer in open fractures of the lower extremity, anastomosis with healthy recipient vessels that have not been affected by the trauma must be performed. Chen et al. [8] recommended using the posterior tibial artery as the recipient vessel, since the anterior tibial artery is injured more frequently than the posterior tibial artery in open fracture of the lower extremity. Several investigators have performed vascular anastomosis distal to the zone of injury, since main vessels in the distal third of the lower extremity pass through a superficial layer [9, 10]. Kolker et al. [11] reported no differences in operative outcomes between use of vascular anastomosis proximal or distal to the zone of injury.

This retrospective study examined recipient vessels and vascular anastomosis techniques in 18 consecutive patients who underwent free flap transfer at an early stage after suffering open fracture of the lower extremity.

## 2. Patients and Methods

We performed free flap transfer within 1 week after injury in 18 consecutive patients (15 men and 3 women) who suffered Gustilo type IIIB open fracture of the lower extremity between January 2002 and December 2008. Mean age at the time of surgery was 31.9 years (range, 18–58 years). The causes of injury were a traffic accident in 15 cases and an occupational accident in 3 cases. Data on fracture site, transferred flaps, vessels selected for anastomosis, anastomosis techniques, and postoperative complications were obtained from medical records. Mean duration of follow-up was 41 months (range, 7–86 months).

## 3. Results

The fracture sites were the proximal third of the lower extremity in 2 cases, the middle third of the lower extremity in 7, and the distal third of the lower extremity in 9. The transferred free flaps were an anterolateral thigh flap in 13 cases and a latissimus dorsi musculocutaneous flap in 5. The anastomosed recipient arteries were the anterior tibial artery in 10 cases, the posterior tibial artery in 6, the popliteal artery in 1, and the superior medial genicular artery in 1.

The vascular anastomosis techniques used were flow-through anastomosis in 12 cases, end-to-side anastomosis in 5, and end-to-end anastomosis in 1. Postoperative complications were congestion due to thrombosis in 2 patients who subsequently underwent reexploration and deep infection that subsided with additional debridement in 1 patient. Free flaps survived in all patients, including the 3 patients who underwent reoperation (Table 1).

## 4. Case Reports

### 4.1. Case 1

**Figure 1:**
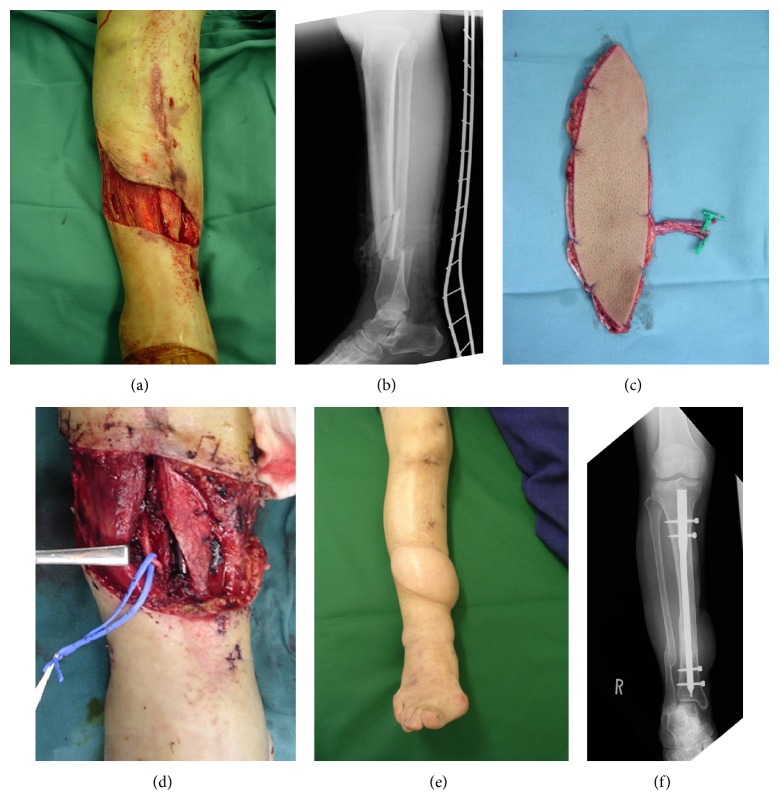
(a) Open fracture of the lower extremity is accompanied by an moderate soft tissue defect on the anterior lower extremity. (b) X-ray findings. (c) Anterolateral thigh flap harvested from the same side. (d) The anterior tibial artery was selected for end-to-side anastomosis. (e) Appearance at 7 months postoperatively. (f) X-ray findings at 7 months postoperatively, showing bone union.

A 58-year-old woman suffered open fracture injury to the lower right extremity in a traffic accident. On the day of injury, debridement and external fixation of the open fracture of the lower extremity were performed. On day 6 after injury, reconstruction was performed using intramedullary fixation and a free anterolateral thigh flap. On preoperative medical examination, the dorsalis pedis artery and posterior tibial artery were palpable. In surgery, the anterior tibial artery was carefully dissected to confirm the absence of injury. End-to-side anastomoses of the lateral circumflex femoral artery and anterior tibial artery with the anterolateral thigh flap were performed to preserve arterial blood flow. The anterolateral thigh flap survived without postoperative complications (Figure 1).

### 4.2. Case 2

**Figure 2:**
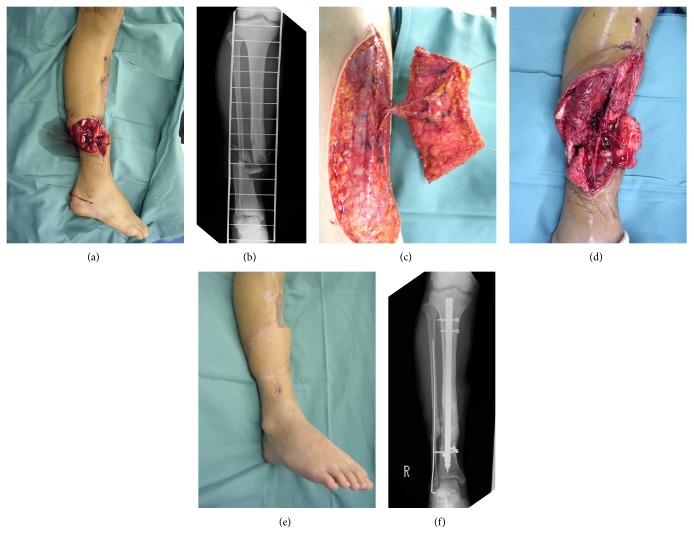
(a) Open fracture is located in the distal third of the lower extremity, accompanied by injury to the anterior tibial artery. (b) X-ray findings. The open fracture is accompanied by a bone defect. (c) The flow-through type anterolateral thigh flap harvested from the same side. (d) The anterior tibial artery is reconstructed by flow-through anastomosis with the lateral circumflex femoral artery. (e) Appearance at 18 months postoperatively. (f) X-ray findings at 18 months postoperatively, showing that union of the bone defect occurred after autologous bone grafting.

A 21-year-old man suffered open fracture injury to the lower right extremity in an occupational accident at a construction site. At the initial surgery, debridement and external fixation were performed. Two days later, open fracture of the lower right extremity was reconstructed with intramedullary fixation and free anterolateral thigh flap. Since the anterior tibial artery had been injured in the open fracture of the lower right extremity, the anterior tibial artery was reconstructed by interposing the lateral circumflex femoral artery of the anterolateral thigh flap. Lateral circumflex femoral veins were anastomosed with the concomitant and great saphenous veins using end-to-end anastomosis. The anterolateral thigh flap survived without postoperative complications. Four months after injury, autologous bone grafting was performed for the bone defect in the open fracture of the lower extremity. Bone union was achieved by 18 months after injury, and the patient has since returned to his original occupation (Figure 2).

## 5. Discussion

The selection of recipient vessels is crucial when reconstructing traumatized lower extremities with free flap. Since the anterior tibial artery is prone to injury in lower extremity trauma, the posterior tibial artery is often selected as the recipient vessel [8]. For recipient vessel selection, in addition to intraoperative examination, preoperative palpation, Doppler flowmetry, and angiography were conducted. Isenberg and Sherman [12] reported that if no problems are seen with the dorsalis pedis artery and posterior tibial artery based on palpation, Doppler flowmetry, and Allen's test, preoperative angiography is unnecessary. Lutz et al. [13] also indicated that preoperative angiography should be applied only when pedal pulses of both the dorsalis pedis and posterior tibial arteries are not palpable and that routine preoperative angiography is unnecessary. On the other hand, Duymaz et al. [14] recommended computed tomography angiography as the first-stage diagnostic procedure, as this method is superior for visualizing the hemodynamics of the traumatized lower extremity. When the dorsalis pedis artery and/or posterior tibial artery are not palpable, we conduct computed tomography angiography to confirm the vascular injury site prior to free flap transfer.

The anterior lower extremity is often injured in open fracture of the lower extremity, and recipient vessels pass through a deeper layer in parts more proximal to the zone of injury, making vascular anastomosis increasingly difficult. For this reason, Stompro and Stevenson [9] conducted free flap transfer with distally based anastomosis for surgery performed in the distal third of the lower extremity, where recipient vessels pass through the superficial layer. Minami et al. [10] also stated that distally based anastomosis is useful in reconstruction of the anterior lower extremity with free flap transfer. Kolker et al. [11] reported that the outcomes of free flap transfer do not differ between distal and proximal anastomosis, stating that distal anastomosis is appropriate when proper hemodynamics are maintained in the zone of injury.

Godina et al. [15] reported a posterior approach to the recipient vessels. Specifically, they described the usefulness of the posterior approach, which can ensure a sufficient surgical field of view and healthy recipient vessels. Park and Eom [16] recommended the superior medial genicular vessels and descending genicular vessels as recipient vessels around the knee. In the two patients who suffered fracture in the proximal third of the lower extremity, recipient arteries were the superior medial genicular artery in 1 patient and the popliteal artery via a posterior approach in the other patient. Only a few recipient vessels around the knee are available for free flap transfers from the proximal third of the lower extremity, and both arteries have proven useful as recipient arteries.

For vascular anastomosis, to preserve the main arterial flow, we fundamentally perform end-to-side anastomosis or flow-through anastomosis. Various outcomes of end-to-side anastomosis have been reported [2, 9, 17, 18]. Godina [17] reported favorable outcomes from end-to-side anastomosis. However, Khouri and Shaw [2] reported that end-to-side anastomosis is prone to thrombosis. Samaha et al. [7] demonstrated a lack of differences in outcomes between end-to-end anastomosis and end-to-side anastomosis and reported that outcomes are influenced by recipient vessel selection and the condition of blood perfusion from distal areas.

To preserve main arterial flow in open fracture of the lower extremity, we perform end-to-side anastomosis if no obvious injuries to the main artery are present and flow-through anastomosis whenever possible if the fracture is accompanied by injuries to the main artery. Koshima et al. [19] reported several advantages of flow-through anastomosis, indicating that the damaged main vessels can be reconstructed simultaneously with large skin defects, while double artery inflow using both ends of the pedicle artery ensures safe blood circulation in the flap, and two concomitant pedicle veins interposed into the damaged recipient concomitant veins can be used as a drainage system in extremities with severe edema. We perform flow-through anastomosis using lateral circumflex femoral vessels of the anterolateral thigh flap and thoracodorsal vessels of the latissimus dorsi musculocutaneous flap. In the acute phase when the influences of scarring are absent, the dissection is relatively easy even in the anterior tibial artery, which is highly likely to be injured. We, therefore, performed anterior tibial artery reconstruction with flow-through anastomosis as much as possible. Free flap with flow-through anastomosis fundamentally entails a flap with stable blood flow. Although 1 patient who underwent flow-through type anterolateral thigh flap developed partial congestion, the flap ultimately survived reexploration. Free flap transfer with flow-through anastomosis does not cause vascular insufficiency as long as the surgery is performed meticulously and the proper recipient vessels are selected. When the dorsal pedis and the posterior tibial arteries are palpable, a flow-through anastomosis is not indicated.

With an open fracture of the lower extremity, we utilize an anterolateral thigh flap with the pedicle descending branch of the lateral femoral circumflex artery for the moderate soft tissue defect and the latissimus dorsi musculocutaneous flap with the pedicle thoracodorsal artery and the serratus branch for the extensive soft tissue defect. These two techniques are long and include a large-caliber pedicle, and reconstruction can be performed with either the anterior or posterior tibial artery. Preparation of recipient vessels is easier during the acute phase when the influences of scarring have not yet manifested. Free flap, which allows flow-through anastomosis, is thus optimal for simultaneous reconstruction of the main vessel injury and soft tissue defect from the middle to distal thirds of the lower extremity.

## 6. Conclusions

When injury to the anterior or posterior tibial artery is suspected in open fracture of the lower extremity, we perform computed tomography angiography to evaluate the arterial injury. In open fracture of the lower extremity without arterial injury, we perform free flap transfer with end-to-side anastomosis to preserve the main vessels. When the arterial injury is present from the middle to distal thirds of the lower extremity in open fracture of the lower extremity, we perform free flap transfer with flow-through anastomosis as much as possible. Free flap transfer with flow-through anastomosis is a useful method that can simultaneously reconstruct soft tissue defects and the main artery.

## Figures and Tables

**Table 1:** Patients summary.

Number	Age	Sex	Fracture site	Free flap	Recipient artery	Anastomotic type	Complications	Result	Comments
1	51	M	Distal	ALT	Anterior tibial a.	Flow-through		Successful	
2	53	M	Distal	ALT	Posterior tibial a.	Flow-through		Successful	
3	31	M	Middle	ALT	Anterior tibial a.	Flow-through		Successful	
4	21	M	Distal	ALT	Anterior tibial a.	Flow-through		Successful	
5	18	M	Proximal	ALT	Medial inferior genicular a.	End-to-end		Successful	
6	34	F	Middle	ALT	Posterior tibial a.	End-to-side	Congestion	Reexploration	Survival
7	25	M	Middle	ALT	Anterior tibial a.	End-to-side		Successful	
8	58	F	Middle	ALT	Anterior tibial a.	Flow-through		Successful	
9	50	M	Middle	LD	Posterior tibial a.	End-to-side		Successful	
10	22	M	Distal	ALT	Anterior tibial a.	Flow-through		Successful	
11	19	M	Distal	ALT	Posterior tibial a.	Flow-through		Successful	
12	33	M	Middle	LD	Anterior tibial a.	Flow-through		Successful	
13	24	M	Middle	LD	Anterior tibial a.	Flow-through		Successful	
14	32	M	Distal	ALT	Posterior tibial a.	End-to-side		Successful	
15	30	M	Proximal	LD	Popliteal a.	End-to-side	Deep infection	Debridement	Survival
16	22	M	Distal	ALT	Posterior tibial a.	Flow-through	Congestion	Reexploration	Survival
17	21	M	Middle	ALT	Anterior tibial a.	Flow-through		Successful	
18	30	F	Middle	LD	Anterior tibial a.	Flow-through		Successful	

ALT: anterolateral thigh flap, LD: latissimus dorsi musculocutaneous flap.
